# Magnetized inulin by Fe_3_O_4_ as a bio-nano adsorbent for treating water contaminated with methyl orange and crystal violet dyes

**DOI:** 10.1038/s41598-022-26652-7

**Published:** 2022-12-20

**Authors:** Kamran Valizadeh, Amir Bateni, Nazanin Sojoodi, Maryam Rostami Ataabadi, Amir Hossein Behroozi, Ali Maleki, Zhenjiang You

**Affiliations:** 1 Department of Chemical Engineering, Science and Research Branch, Islamic Azad University, Tehran, Iran; 2 Department of Chemical Engineering, South Tehran Branch, Islamic Azad University, Tehran, Iran; 3 School of Chemical, Petroleum and Gas Engineering, Iran University of Science and Technology, Tehran, Iran; 4 Catalysts and Organic Synthesis Research Laboratory, Department of Chemistry, Iran University of Science and Technology, Tehran, 16846-13114 Iran; 5 Center for Sustainable Energy and Resources, Edith Cowan University, Joondalup, WA 6027 Australia; 6 School of Chemical Engineering, The University of Queensland, Brisbane, QLD 4072 Australia

**Keywords:** Environmental sciences, Chemistry, Materials science, Nanoscience and technology

## Abstract

Current work focuses on fabricating a new bio-nano adsorbent of Fe_3_O_4_@inulin nanocomposite via an in-situ co-precipitation procedure to adsorb methyl orange (MO) and crystal violet (CV) dyes from aqueous solutions. Different physical characterization analyses verified the successful fabrication of the magnetic nanocomposite. The adsorbent performance in dye removal was evaluated by varying initial dye concentration, adsorbent dosage, pH and temperature in 5110 mg/L, 0.10.8 g/L, 111 and 283–338 K, respectively. Due to the pH of zero point of charge and intrinsic properties of dyes, the optimum pHs were 5 and 7 for MO and CV adsorption, respectively. The correlation of coefficient (*R*^2^) and reduced chi-squared value were the criteria in order to select the best isotherm and kinetics models. The Langmuir model illustrated a better fit for the adsorption data for both dyes, demonstrating the maximum adsorption capacity of 276.26 and 223.57 mg/g at 338 K for MO and CV, respectively. As well, the pseudo-second-order model showed a better fitness for kinetics data compared to the pseudo-first-order and Elovich models. The thermodynamic parameters exhibited that the dye adsorption process is endothermic and spontaneous, which supported the enhanced adsorption rate by increasing temperature. Moreover, the nanocomposite presented outstanding capacity and stability after 6 successive cycles by retaining more than 87% of its initial dye removal efficiency. Overall, the magnetized inulin with Fe_3_O_4_ could be a competent adsorbent for eliminating anionic and cationic dyes from water.

## Introduction

Rapid industrialization and growing human activities have prompted severe environmental concerns worldwide, notably water pollution. Usually, various organic and inorganic contaminants end up in water bodies from different industrial sectors every day^1,2^. Predominantly, textile, plastic, cosmetic and paper production industries continually release toxic dye contaminants into surface waters, negatively impacting the environment, humans and animals^3^. Many commercial dyes are available worldwide, exceeding 10,000 specimens, producing approximately 7 × 10^5^ metric tons of dyes per year^4^. The significant contribution of environmental pollution by dyes is related to industrial wastewater by 20%; only about 12% of dyes are wastes from manufacturing and handling processes. The current critical concern is that a significant portion of effluents containing dyes gets discharged to the adjacent water sources without any treatment^5^.

The presence of such dyes in water is not favorable because they decrease the sunlight penetration through water, reducing the photosynthesis mechanism of aquatic plants. Moreover, these dyes are detrimental to living organisms because of various toxic elements, including aromatics, chloride, and heavy metals^6^. Even in a trace amount, the water-containing dyes could create several health risks in humans, such as cancer, skin problems, and allergic dermatitis^7^. Methyl orange (MO) is a common water-soluble organic synthetic, azo-anionic dye, which is highly carcinogenic, teratogenic, and harmful to organisms and the environment. Also, crystal violet (CV) is a highly toxic and triphenylmethane cationic dye. Its existence in water can cause severe health and environmental problems due to its potent clastogenic nature. Thus, the water contaminated with such dyes should be treated instantaneously worldwide. However, it is not a simple and easy process because of their non-biodegradability, thermal and photostability^8,9^.

Wastewater-containing dyes has been treated by chemical, biological, and physical treatment techniques, including membrane filtration^10^, coagulation^11^, electrodialysis^12^, photocatalytic degradation^13^, adsorption^14^, and chemical oxidation^15^. Among them, adsorption has received much attention due to its superior features. The ease of operation, cost-effectiveness, low operating temperature, no secondary pollution, simplicity, and simultaneous removal of pollutants are great examples of adsorption benefits compared to other methods^16^. Various adsorbents, such as carbonaceous materials, have been used for this purpose thus far. However, their applications in treating dye-polluted water are usually restricted due to their high regeneration cost, low sorption efficiency, and complicated separation process^17^.

In recent three years, several researchers have attempted to develop new chitosan-based bio-nanocomposites magnetized with metal oxides as sustainable adsorbents for water and wastewater treatment^18^. Jawad et al.^19^ fabricated a new hybrid bio-adsorbent of crosslinked chitosan-epichlorohydrin/TiO_2_ nanocomposite for removing reactive red 120 dye from an aqueous solution, achieving the maximum adsorption capacity of 210 mg/g at 30C. They found that electrostatic attraction, H-bonding, and n-π stacking are responsible for the adsorption of dye molecules on the adsorbent surface. Reghioua et al.^20^ synthesized a Schiff’s base chitosan-glutaraldehyde composite magnetized by Fe_3_O_4_ and ZnO to remove Remazol brilliant blue R dye from wastewater. The highest capacity was found to be 176.6 mg/g at 60 °C by incorporating 25% ZnO into the nanocomposite structure. Kazemi and Javanbakht^21^ fabricated a magnetic zeolite/chitosan/alginate nanocomposite crosslinked by CaCl_2_ and glutaraldehyde to enhance its stability. The results indicated a spontaneous exothermic mechanism of methylene blue adsorption by the synthetic adsorbent.

Al-Musawi et al.^22^ coated a chitosan polymer with magnetite Fe_2_O_3_ nanoparticles to adsorb acid blue 113 dye from an aqueous solution under various experimental factors, such as initial dye concentration, solution pH, nanocomposite dosage, adsorption time and temperature. They found a maximum uptake capacity and dye removal efficiency of 128 mg/g and 99.63% under the optimum conditions, respectively. Tanhaei et al.^23^ synthesized a chitosan-based adsorbent magnetized with carbon-Fe_3_O_4_ core–shell nanoparticles for methyl orange removal from aqueous solutions with a maximum uptake capacity of 425 mg/g at 45 °C. Neves et al.^24^ magnetized chitosan with a novel graphene oxide derivative, grafted long-chain quaternary ammonium salt with arenediazonium salts, to improve its amphiphilic feature and expand the adsorption spectrum. The magnetized sorbent obtained a maximum dye removal capacity and rate of 650 mg/g and 95%, respectively. Their magnetic composite exhibited the potential of being a filtering agent with simple removal from aqueous solutions via the magnet field after each use for removing basic brown 4.

Recently, Hernández-Martínez et al.^25^ reported that inulin could be a suitable replacement for chitosan, particularly for removing Pb^2+^ from wastewater, to enhance the scalability and production cost of polyurethane based-nanocomposite fabrication. Inulin is a combination of linear fructose polymers with various chain lengths and a glucose molecule at every C2 end, belonging to the fructan group of polysaccharides^26^. As a Generally Recognized as Safe (GRAS) substance, inulin can be produced by a host of microorganisms, which can be extensively detected in nature as a storage carbohydrate. Inulin is commercially made from chicory and partially dissolved in water, enabling it to add to aqueous media without precipitation^27^. Figure S1 (Supplementary) illustrates the general production process of inulin from chicory.

This work aims to fabricate a new biocompatible adsorbent of Fe_3_O_4_@inulin nanocomposite for removing cationic and anionic dyes from water for the first time. The prepared adsorbent was characterized by various analyses, such as scanning electron microscopy (SEM) with energy dispersive X-Ray analysis (EDX), X-ray powder diffraction (XRD), Fourier-transform infrared spectroscopy (FTIR), vibrating sample magnetometry (VSM), Brunauer–Emmett–Teller (BET), and thermogravimetric analysis (TGA). Moreover, kinetics and isotherm studies assessed the adsorption of MO and CV dyes with the synthesized adsorbent. Finally, the adsorbent reusability was checked after several cycles of reusing.

## Materials and methods

### Materials

**Figure 1:**
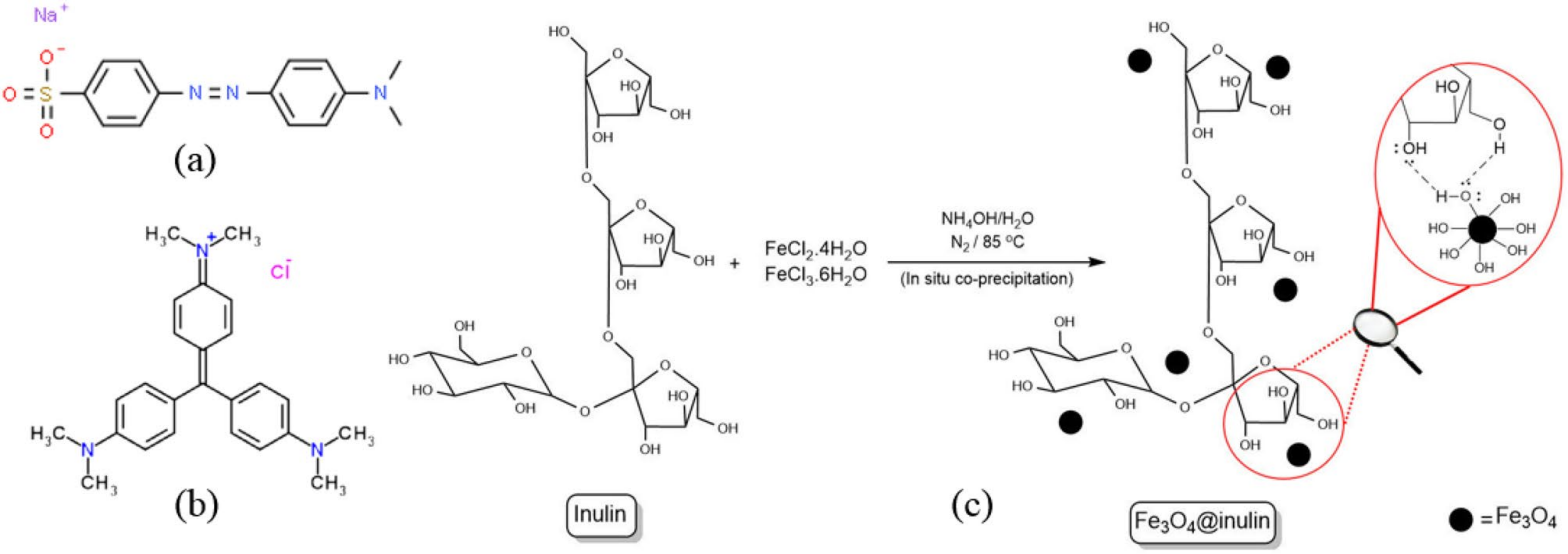
Molecular structures of (**a**) methyl orange, (**b**) crystal violet, and (**c**) Fe_3_O_4_@inulin with synthesis procedure.

Iron (II) chloride tetrahydrate (FeCl_2_⋅4H_2_O (aq)), Iron (III) chloride hexahydrate (FeCl_3_⋅6H_2_O (aq)), ammonia (NH_3_, 25%), hydrochloric acid (HCl, 0.1 M), sodium hydroxide (NaOH, 0.1 M), inulin from chicory, methyl orange and crystal violet dyes were purchased from Sigma-Aldrich company. All materials were used in their analytical grades without any further purification. The molecular structures of methyl orange and crystal violet as dye contaminants are depicted in Fig. 1a,b, respectively.

### Synthesis of Fe_3_O_4_@inulin

First, 60 mL of deionized water was added to a 250 mL two-mouth glass flask. The amount of 0.7 g of FeCl_2_⋅4H_2_O was poured into the flask under stirring, and then the amount of 1.9 g of FeCl_3_⋅6H_2_O was added to the solution. The mixture was stirred for 5 min until completely dissolved to obtain a uniform solution. Next, 0.75 g of inulin was slowly added to the solution in several steps under stirring for 25 min. The ambient temperature was raised to 85 °C under a nitrogen atmosphere, and 10 mL of NH_3_OH was injected dropwise by syringe for 70 min. The color of the solution turned black, indicating the formation of Fe_3_O_4_ nanoparticles during the injection. The mixture was further stirred for 30 min to allow the remaining NH_3_OH to react. Next, the magnetized inulin nanoparticles were separated from the solution with a magnet and washed with deionized water four times to remove the unreacted materials from the nanocomposite. The magnetized inulin was transferred to an oven and placed at 55 °C for 15 h to dry. Finally, the powdered nanoparticles were obtained for the experiments and characterization. Figure 1c illustrates the molecular scheme of the synthesis procedure of Fe_3_O_4_@inlulin.

### Characterization

The crystal network of the synthesized adsorbent nanoparticles was examined by an XRD analysis (STOE STADI-MP model, Germany) with Cu Ka radiation (= 1.54 Å) and 2θ of 10°–80°. The adsorbent functional groups were evaluated using the FTIR analysis (Spectrum Rx1 model, Perkin Elmer Co.) in 400–4000 cm^−1^. The magnetization property of the prepared nanocomposite was assessed by a VSM device (LBKFB model, Iran) under the ambient condition and in the range of − 4000 to 4000 Oe. The structure, morphology and elemental distribution of the adsorbent were identified by SEM–EDX analysis (MIRA3 TESCAN). By heating the sample at 120 °C to eliminate any impurities in its structure, it was analyzed by BET analysis (Micromeritics, Model ASAP 2020, USA) at 77 K. Several NaNO_3_ solutions (0.1 M) with 50 mL volume were added to different flasks at the ambient condition to measure the point of zero charges (PZC). The initial pH values (pH_i_) were adjusted by adding HCl and NaOH solutions determined by a pH meter (Crison, Spain). 2 g of sample was added to each solution and then stirred for 48 h under ambient conditions. The final pH values (pH_f_) can be measured to calculate the pH of zero point of charge (pH_ZPC_).

### Adsorption experiments

The dye removal experiments have been conducted to determine the adsorption performance of synthesized magnetic nanocomposites as follows. The batch adsorption was carried out in an Erlenmeyer flask with a volume of 250 mL by adding 0.1–0.8 g of the magnetic bio-nano adsorbent in the controlled operational conditions for the isotherm and kinetics investigations. The initial concentration (*C*_*dye*_) of MO and CV varied in the range of 25–200 mg/L. The volume of every solution was 100 mL, and the adsorption temperature was changed from 10 to 65C. The solution pH was considered in the range of 1–11 to find the best conditions for each dye removal. To do so, the calibration curves were first plotted for both dyes at different pHs. Next, the adsorption experiments were conducted at different pHs and various adsorption times (15–180 min, every 15 min sampling). Finally, the calibration curve with the pH proportional to the corresponding solution pH was used to measure the concentration of the remaining dye in the aqueous solution. The dye concentration was determined by UV–Vis spectrophotometer (Rayleigh/UV 2601) at a wavelength of 464 nm and 590 nm for MO and CV, respectively. The formulas of the removal efficiency and adsorption capacity (*Q*) are as follows^28^:

1 $${\text{Removal }}\,{(}\% ) = \frac{{C_{0} - C_{e} }}{{C_{0} }} \times 100$$

2 $$Q\,{\text{ (mg/g)}} = \frac{{\left( {C_{0} - C_{e} } \right)V}}{m}$$

in which, *C*_0_ and *C*_*e*_ (mg/L) are the initial and equilibrium concentrations of dyes in the aqueous solution, respectively. The terms *m* (g) and *V* (L) are the mass of the nanocomposite and the volume of the aqueous solution, respectively. Each experimental run was repeated three times, and all reported results are the averaged data with a deviation error of ± 5%.

## Results and discussion

### Adsorbent characterization

**Figure 2:**
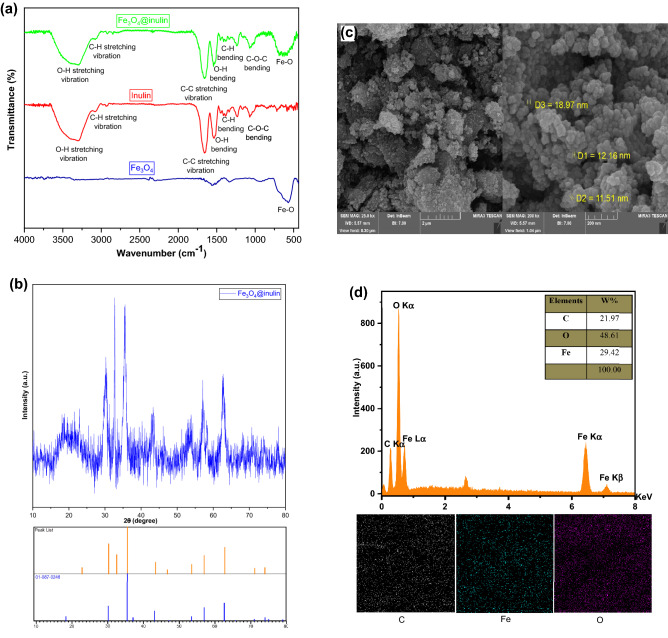
(**a**) FTIR spectra of Fe_3_O_4_, inulin, and Fe_3_O_4_@inulin; (**b**) XRD pattern of the prepared Fe_3_O_4_@inulin nanocomposite; (**c**) SEM images and (d) EDX results of the synthesized Fe_3_O_4_@inulin bio-nano adsorbent.

FTIR analysis is a practical tool for detecting the functional groups in the structure of materials. Figure 2a presents the FTIR spectra of Fe_3_O_4_, inulin, and Fe_3_O_4_@inulin compounds. In the Fe_3_O_4_ spectrum, there is a prominent peak related to the FeO bond at 578 cm^−1^^29^. The FTIR spectra of inulin and Fe_3_O_4_@inulin nanocomposite illustrate several similar adsorption bands: broad adsorption bands at 3428 and 1531 cm^−1^ are attributed to the bending and stretching vibration of the OH bond, respectively^30^, which is abundant in the inulin backbone. The adsorption band at about 3095 cm^−1^ is associated with the stretching vibration of the CH bond^31^. The peak at 1101 cm^−1^ is related to the asymmetric stretching vibration of the COC bond, and an absorption band at 1052 cm^−1^ is attributed to the stretching vibration of the CO bond^32^. Furthermore, the appearance of FeO peak in the Fe_3_O_4_@inulin spectrum confirms the successful fabrication of the nanocomposite.

The crystalline structure of Fe_3_O_4_@inulin nanocomposite studied by XRD analysis is shown in Fig. 2b. The individual weak broad peak around 15°–23° indicates the amorphous phase of inulin. According to the standard patterns of Fe_3_O_4_ in card no. JCPDS, 01-087-0246, this magnetic material has some broad characteristic diffraction peaks at 2θ of 18.27°, 30.07°, 35.46°, 37.13°, 43.02°, 57.16°, 62.74°, 71.21°, and 73.49°^32^. Sharp and broad peaks demonstrate the crystalline and amorphous areas in the Fe_3_O_4_@inulin nanocomposite, respectively. Based on Scherer's equation^33^, the average crystalline size for Fe_3_O_4_@inulin bio-nano adsorbent is 10.9 nm.

SEM–EDX analysis of the Fe_3_O_4_@inulin adsorbent has been conducted to evaluate its morphology and elemental distribution, as presented in Fig. 2c,d. As shown in Fig. 2c at low magnification, the surface morphology of Fe_3_O_4_@inulin nanocomposite is rough, heterogeneous and irregular with some cavities. However, according to the SEM images at higher magnifications, the adsorbent surface depicts a relatively uniform, regular distribution of spherical nanoparticles on the Fe_3_O_4_@inulin nanocomposite surface, with a particle diameter range of 10–20 nm. Moreover, the elemental mapping in Fig. 2d illustrates a uniform distribution of all elements, including carbon, oxygen and iron, within the adsorbent structure, exhibiting the maximum content of oxygen with a contribution of 48.61%.

**Figure 3:**
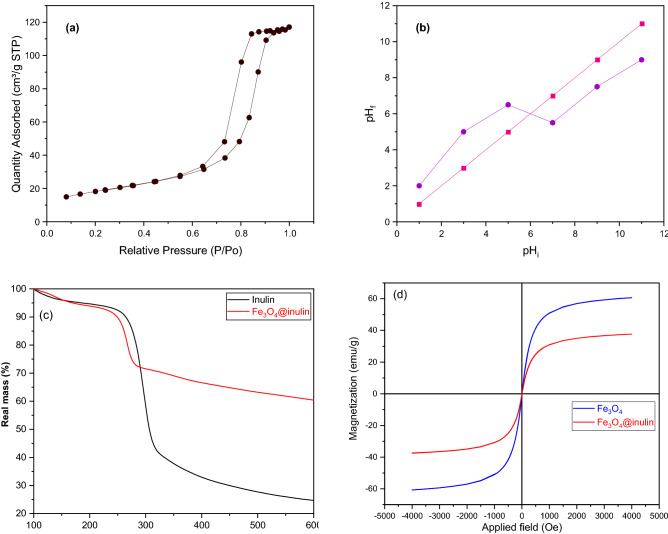
(**a**) N_2_ adsorption/desorption curve of the Fe_3_O_4_@inulin adsorbent; (**b**) pH_f_ versus pH_i_ for determining pH_ZPC_ of the Fe_3_O_4_@inulin nanocomposite; (**c**) TGA curves of inulin and Fe_3_O_4_@inulin; (**d**) magnetic hysteresis curves of Fe_3_O_4_ and Fe_3_O_4_@inulin.

N_2_ adsorption/desorption isotherm of the synthesized Fe_3_O_4_@inulin nanoparticles is shown in Fig. 3a, resulting from the BET analysis. This curve exhibits the mesoporous structure of the adsorbent supported by the type IV isotherm based on the IUPAC classification^34^. Such mesopores are favorable for enhancing the specific surface area of the material. According to the calculations by the BJH method^35^, the specific surface area of 66.15 m^2^/g, pore volume of 0.181 cm^3^/g, and average pore diameter of 11.027 nm are obtained for Fe_3_O_4_@inulin adsorbent. The factor pH_PZC_ shows the charge of the adsorbent surface, the pH value where the net surface charge is zero. The pH_f_ against pH_i_ values are plotted in Fig. 3b to determine this factor for the prepared Fe_3_O_4_@inulin composite, indicating a pH_PZC_ of approximately 6. Therefore, the adsorbent surface will be negatively charged at pH values higher than 6, while it will be positively charged otherwise.

The TGA method has been used to assess the thermal resistance of magnetic Fe_3_O_4_@inulin in the temperature range of 100–600 °C under an air atmosphere. Figure 3c illustrates the weight loss curve of pure inulin and Fe_3_O_4_@inulin materials. Both substances have initially experienced a weight loss of about 9% due to the elimination of free water and moisture evaporation. The most significant decomposition rate related to inulin exists in the range of 240–310 °C with a reduction in its weight by about 50%, which might be associated with dehydration. However, the most considerable weight loss of the Fe_3_O_4_@inulin nanocomposite occurs at 240–285 °C with a reduction of approximately 20%, representing the primary degradation of magnetic composite possibly caused by selective dehydration. The residual weights until 600 °C for inulin and Fe_3_O_4_@inulin are about 25% and 65%, respectively. As a result, adding magnetic Fe_3_O_4_ nanoparticles into the inulin matrix can significantly increase its thermal stability due to strong intermolecular interactions. The magnetic characteristic of magnetized inulin is evaluated by VSM analysis. Figure 3d depicts the magnetic hysteresis loop of Fe_3_O_4_ and Fe_3_O_4_@inulin nanoparticles. The magnetic saturation of Fe_3_O_4_@inulin nanocomposite is 40.21 emu/g, which is 34% lower than that of Fe_3_O_4_ nanoparticles. This lower saturation is due to the presence of non-magnetic inulin, i.e., inulin, in the structure of the synthesized composite. However, this slight decrease in the magnetic saturation is reasonable, and the nanocomposite can still be removed from the reaction solution efficiently by applying an external magnetic field or a simple magnet.

### Effect of operating conditions

**Figure 4:**
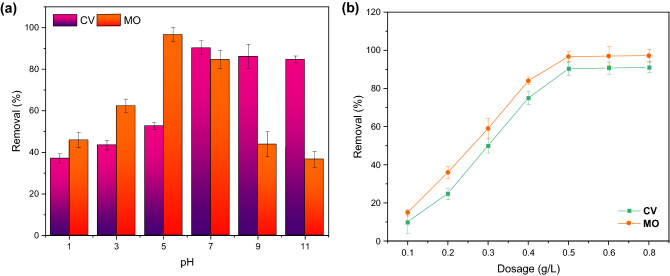
(**a**) Effect of solution pH (*C*_*a*_ = 0.5 g/L, *C*_*dye*_ = 25 mg/L); (**b**) Effect of adsorbent dosage (optimum pH = 7 for CV and 5 for MO) on the dye removal efficiency; T = 25 °C, t = 180 min.

The adsorption experiments were performed at different pHs (1–11) to find the best solution pH for each dye. The other operating factors were constant, including the temperature of 25 °C, the adsorbent dosage of 0.5 g/L, the dye concentration of 25 mg/L, and the adsorption time of 180 min. As known, molecular aggregation occurs in aqueous solutions when the pH value is very low, resulting in considerable aggregates; thus, it is reasonable to detect the optimum pH for every solution. Figure 4a shows the removal efficiency of both dyes at different solution pHs. The maximum adsorption rates of about 95% and 91% were obtained at a pH of 5 and 7 for MO and CV dyes, respectively. Such adsorption trends by pH variation can be explained by the pH_ZPC_ curve, reported in Fig. 3b. The solution pH affects the net surface for amphoteric molecules containing both negative and positive charges. They become more positively or negatively charged by either gaining or losing protons. Because MO acts as a cationic dye in highly acidic solutions^36^, its removal efficiency is lower due to the electrostatic repulsion between cationic dye and negatively charged adsorbent surface. By further increasing the solution pH, the number of positive charges is reduced, achieving the maximum MO removal efficiency at pH 5. However, at a pH higher than 5, the excessive quantity of hydroxyl ions competing with MO molecules for adsorption sites and electrostatic repulsion will reduce its adsorption rate. Moreover, the color of the solution containing MO dye becomes shallow, not only because of the change in pH but also due to the association with the adsorption by the Fe_3_O_4_@inulin adsorbent. For CV adsorption, lower removal efficiency at an acidic pH might be due to excessive H^+^ ions competing with CV dye cations for the adsorption sites^37^. Under a pH higher than the neutral solution, the CV removal efficiency does not change significantly. Therefore, the optimal pH value for CV removal was found to be 7.

Figure 4b exhibits the effect of Fe_3_O_4_@inulin adsorbent dosage on the removal efficiency of both dyes under different concentrations in the range of 0.10.8 g/L. The optimal solution pH related to each dye was considered in this case study. The removal efficiencies by 0.5 g/L concentration of adsorbent are about 6.3 and 9.1 times when the adsorbent dosage is 0.1 g/L for MO and CV adsorption, respectively. This significant increment is due to the enhanced active functional groups and increased number of active binding sites in the nanocomposite. At dosages higher than 0.5 g/L, the removal efficiency increases insignificantly because of the saturation of adsorption sites. Hence, the optimum amount of adsorbent for adsorbing both dyes is selected as 0.5 g/L for the following examinations.

### Adsorption isotherms

Adsorption isotherm models can achieve the equilibrium relations between the adsorbate and adsorbent. To estimate the adsorption behavior of the prepared nanocomposite, four non-linear isotherm models, Langmuir, Freundlich, Dubinin–Radushkevich (D–R), Temkin utilized, and their correlations are given below^38^:

3 $${\text{Langmuir:}}\quad \, {Q}_{e} = \frac{{{Q}_{m} K_{L} C_{e} }}{{1 + K_{L} C_{e} }}$$

4 $${\text{Freundlich:}}\quad \, {Q}_{e} = K_{F} C_{e}^{\frac{1}{n}}$$

5 $${\text{Dubinin{-}Radushkevich:}}\quad \, {Q}_{e} = {Q}_{m} \exp \left( { - K_{D} \left( {RT\ln \left( {1 + \frac{1}{{C_{e} }}} \right)} \right)^{2} } \right)$$

6 $${\text{Temkin:}}\quad \, {Q}_{e} = \frac{RT}{{B_{T} }}\ln (K_{T} C_{e} )$$

where ${Q}_{e}$ is the amount of dye adsorbed at the equilibrium state, ${Q}_{m}$ is the Langmuir monolayer adsorption capacity, *K*_*L*_ is the Langmuir isotherm constant, *K*_*F*_ is the Freundlich isotherm constant, and *n* is the Freundlich exponent^39^. The term *K*_*D*_ is the isotherm constant in the thermodynamic equilibrium constant in the adsorption process, *R* is the gas constant (8.314 J/mol K), and *T* is the temperature. Also, the terms *K*_*T*_ and *B*_*T*_ are the Temkin isotherm constant and a constant related to the heat of adsorption, respectively. The Langmuir isotherm is typically utilized when there is ideal monolayer adsorption on a homogeneous surface. The Freundlich isotherm is generally appropriate for nonideal adsorption on heterogeneous surfaces. The Freundlich isotherm is a purely empirical model, presuming that a great number and a variety of existing sites act simultaneously, each with a different free energy of sorption. The D-R model assumes that the adsorption equilibrium relation for a specific adsorbent-adsorbate combination can be expressed by the adsorption potential independent of temperature. The Temkin isotherm model presumes that the adsorption heat of all molecules is linearly reduced with the increment in coverage of the adsorbent surface and that adsorption is described by a uniform distribution of binding energies^40^.

**Figure 5:**
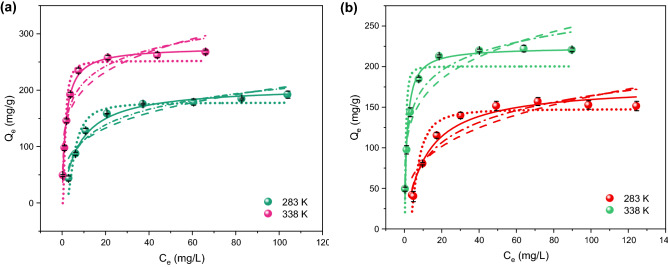
Isotherm curves under different temperatures for (**a**) MO adsorption at pH of 5 and (**b**) CV adsorption at pH of 7; solid lines: Langmuir, dashed lines: Freundlich, dotted lines: Dubinin-Radushkevich, dash-dotted lines: Temkin.

The isotherm modeling of MO and CV adsorption onto the Fe_3_O_4_@inulin composite is illustrated in Fig. 5 for two temperatures of 283 and 338 K. The results and fitting parameters related to all isotherm models at other temperatures within this range are reported in Table 1. In this study, the adsorbent dosage and adsorption time are 0.5 g/L and 180 min, respectively. At all temperatures, the Langmuir isotherm model exhibited a better prediction for the adsorption data related to both dyes due to a coefficient of determination (*R*^2^) higher than 0.98 and the minimum reduced chi-squared value among all considered models. The temperature rise leads to an increase in the adsorption capacity of the nanocomposite, indicating that higher temperatures favor the MO and CV adsorption process because of its endothermic nature. Also, in the Freundlich isotherm model, the constant (*K*_*F*_) value is higher at elevated temperatures, further demonstrating that the adsorption process is endothermic. This increment in adsorption rate can be explained by the temperature-induced breakage of some internal bonds at the composite active surface, leading to an enhanced number of adsorption sites, as well as the dye molecule diffusion into the composite surface cavities raised by a temperature trigger. Additionally, the values of 1/*n* in the Freundlich models at all temperatures are smaller than 1, demonstrating a favorable adsorption process.

**Table 1:** Fitting parameters obtained from isotherm models for the MO and CV adsorption onto the Fe_3_O_4_@inulin nanocomposite.

Model	Dye	MO				CV			
	Temperature [K]	283	298	318	338	283	298	318	338
Langmuir	*K*_*L*_ [L/mg]	0.1289	0.2296	0.2869	0.6137	0.0902	0.1586	0.3079	0.7581
	*Q*_*m*_ [mg/g]	207.42	223.64	255.23	276.26	177.14	192.88	209.95	223.74
	Reduced chi-squared	59.749	49.023	36.121	52.880	83.947	46.669	17.638	85.860
	*R*^2^	0.9819	0.9888	0.9941	0.9934	0.9683	0.9868	0.9962	0.9860
	Adjusted *R*^2^	0.9789	0.9870	0.9931	0.9923	0.9638	0.9849	0.9957	0.9840
Freundlich	*K*_*F*_ [(mg/g)(L/mg)^1/n^]	59.477	79.173	93.948	125.571	41.901	57.730	77.454	102.27
	*n* [dimensionless]	3.732	4.198	4.184	4.872	3.384	3.829	4.353	5.061
	Reduced chi-squared	471.78	414.04	764.35	1184.01	452.68	528.32	699.24	693.89
	*R*^2^	0.8572	0.9069	0.8759	0.8523	0.8296	0.8508	0.8621	0.8869
	Adjusted *R*^2^	0.8334	0.8902	0.8552	0.8277	0.8062	0.8296	0.8310	0.8708
Dubinin–Radushkevich	*K*_*D*_	4.8122	2.1686	1.2620	3.1610	6.9059	2.0001	5.5347	1.0984
	*Q*_*m*_	178.47	199.62	228.64	251.93	147.84	164.55	184.23	200.33
	Reduced chi-squared	233.84	666.68	605.74	730.41	186.25	418.77	516.45	966.34
	*R*^2^	0.9292	0.8484	0.9016	0.9089	0.9298	0.8817	0.8907	0.8425
	Adjusted *R*^2^	0.9174	0.8232	0.8852	0.8937	0.9198	0.8648	0.8751	0.8200
Temkin	*B*_*T*_	59.182	66.753	61.144	67.504	66.476	70.141	75.979	88.877
	*K*_*T*_	1.6319	4.8942	5.3783	16.962	0.9660	2.2236	5.5592	24.041
	Reduced chi-squared	218.68	138.69	289.24	492.37	220.70	235.52	297.04	229.57
	*R*^2^	0.9338	0.9684	0.9530	0.9386	0.9169	0.9335	0.9371	0.9626
	Adjusted *R*^2^	0.9227	0.9632	0.9452	0.9283	0.9050	0.9240	0.9281	0.9572

### Adsorption kinetics

Kinetics investigation for the pollutant adsorption from contaminated water can be worthwhile because it enables finding the adsorption equilibrium time, adsorption kinetics rate, and the adsorbate concentration in each phase after achieving the equilibrium state. Here, pseudo-first-order (PFO), pseudo-second-order (PSO), and Elovich kinetics models are used to better examine the adsorption mechanism of MO and CV dyes onto the Fe_3_O_4_@inulin nanocomposite.

**Figure 6:**
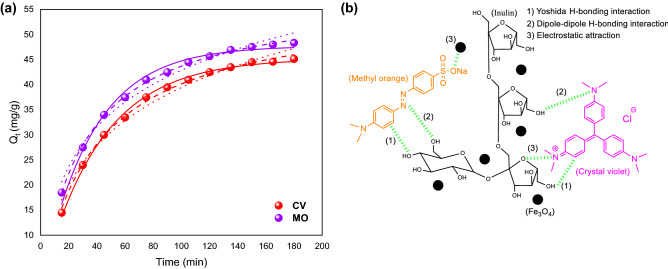
(**a**) Kinetics data of MO and CV adsorption process at their optimum solution pH predicted by non-linear kinetics models at *C*_*a*_ = 0.5 g/L and *C*_*dye*_ = 25 mg/L: solid line: PFO, dashed line: PSO, dotted line: Elovich; (**b**) proposed mechanism of dye adsorption onto the Fe_3_O_4_@inulin nanocomposite.

The PFO model assumes that the rate of variation in solute adsorption is directly proportional to the difference in saturated concentration and the amount of adsorptive solid adsorbed over time. The PSO kinetics describes that the adsorption rate is measured by the interaction between the adsorbate and adsorbent species. The Elovich model can estimate the surface and mass diffusion, activation and deactivation energy of the adsorption system. In this model, the adsorption rate of solute is exponentially reduced by increasing the adsorbed solute amount^41^. The non-linear equations of these models are as follows^42^:

7 $${\text{PFO:}}\quad \, {Q}_{t} = Q_{e} (1 - \exp ( - k_{1} t))$$

8 $${\text{PSO:}}\quad \, {Q}_{t} = \frac{{k_{2} {Q}_{e}^{2} t}}{{1 + {Q}_{e} k_{2} t}}$$

9 $${\text{Elovich:}}\quad {Q}_{t} = \frac{1}{\beta }\ln (\alpha \beta t + 1)$$

where ${Q}_{t}$ is the amount of dye adsorbed at time *t*, and the terms *k*_1_ and *k*_2_ are the rate constants for PFO and PSO models, respectively. Also, the terms and are the initial adsorption rate and the desorption constant in the Elovich model, respectively. The fitting results of kinetic data are shown in Fig. 6. Among these three models, the PSO model provides excellent predictions of the experimental kinetics data of the adsorption capacity of the prepared nanocomposite for both dyes. Table 2 presents the parameters resulting from fitting the kinetics models to the experimental data. The highest *R*^2^ and lowest reduced chi-squared values confirm that the adsorption mechanism can be characterized by a PSO kinetics model. The applicability of this model implies that the adsorption of both dyes onto the Fe_3_O_4_@inulin adsorbent surface is a chemisorption process^43^, where the molecules of dyes have been bounded to the Fe_3_O_4_@inulin through surface exchange reactions. The dye adsorption mechanism by the prepared Fe_3_O_4_@inulin nanocomposite is also shown in Fig. 6b. The most contributive forces are electrostatic attraction and hydrogen bonding interactions (dipole–dipole and Yoshida). The contribution of each force in dye adsorption differs at different pHs, as its details are reported in the literature^44^.

**Table 2:** Fitting parameters obtained from kinetics models for MO and CV adsorption onto the Fe_3_O_4_@inulin nanocomposite.

Parameter	Dye	
	MO	CV
**PFO**		
*Q*_*e*_	47.736	45.243
*k*_1_ [min^−1^]	0.0276	0.0239
Reduced chi-squared	1.148	0.3634
*R*^2^	0.9880	0.9964
Adjusted *R*^2^	0.9868	0.9960
**PSO**		
*Q*_*e*_	57.523	56.094
*k*_2_ [g mg^−1^ min^−1^]	5.501	4.497
Reduced chi-squared	0.1265	0.3192
*R*^2^	0.9986	0.9968
Adjusted *R*^2^	0.9985	0.9965
**Elovich**		
*α* [mg/g min]	3.344	2.233
*β*	0.0764	0.0719
Reduced chi-squared	1.7776	2.0849
*R*^2^	0.9814	0.9793
Adjusted *R*^2^	0.9796	0.9773

### Thermodynamic study

The thermodynamic parameters of the enthalpy (ΔH°), entropy (ΔS°), and Gibbs free energy (ΔG°) were measured by the following correlations:

10 $$K_{c} = \frac{{{Q}_{e} }}{{C_{e} }}$$

11 $$\Delta G^{ \circ } = - RT\ln K_{c}$$

12 $$\ln K_{c} = - \frac{{\Delta H^{ \circ } }}{RT} + \frac{{\Delta S^{ \circ } }}{R}$$

where *K*_*c*_ is the adsorption equilibrium constant. The thermodynamic data calculated from the above equations are reported in Table 3. The positive value of ΔH° proposed that the adsorption process was endothermic, which agreed with the increasing dye adsorption with temperature. The ΔG° with negative values indicated a spontaneous adsorption process. The ΔG° is reduced by increasing temperature, proposing that the adsorption process is more favorable at higher temperatures. Additionally, the ΔS° with positive quantities demonstrated enhanced randomness at the solution-solid interface because of several structural changes throughout the process. The surface of the composites and dye molecules in the aqueous solution was surrounded by hydration layers of water molecules. Throughout the adsorption process, the water molecules ordered in these hydration layers were disturbed and compelled, improving the degree of freedom in the adsorbent-dye interaction.

**Table 3:** Thermodynamic parameters for CO_2_ adsorption onto Fe_3_O_4_@inulin nanocomposite.

Dye\parameter	ΔH° [kJ/mol] at 298 K	ΔS° [kJ/mol K] at 298 K	ΔG° [kJ/mol]			
			283 K	298 K	313 K	338 K
MO	11.5448	46.0172	− 1.4519	− 2.1345	− 2.9897	− 3.9379
CV	10.2271	37.8977	− 0.4691	− 1.0695	− 1.7101	− 2.5345

### Adsorbent reusability

**Figure 7:**
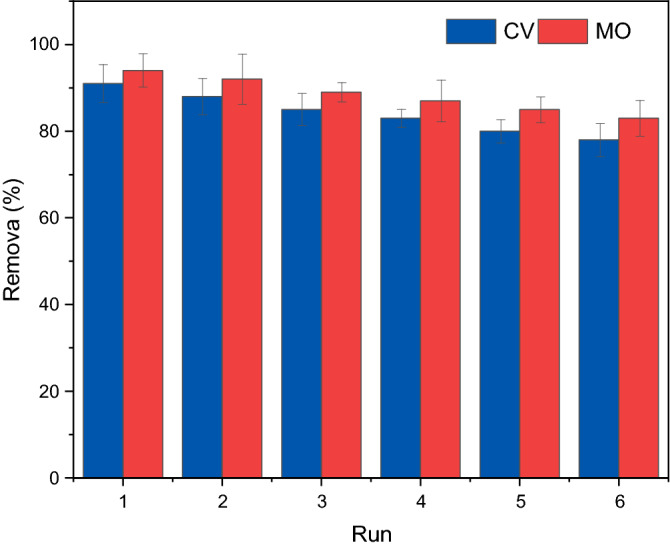
Adsorbent reusability for MO and CV adsorption by Fe_3_O_4_@inulin in terms of removal efficiency; T = 298 K, pH = 7 for CV and 5 for MO, *C*_*a*_ = 0.5 g/L, *C*_*dye*_ = 25 mg/L, t = 180 min.

In addition to acceptable adsorption performance, easy reusability and multi-cycle utilization are critical factors in providing a feasible, scalable and efficient treatment system because it influences the adsorbent performance and reduces the operational cost. The ethanol and acetone with high dipole moments were harnessed as the desorption agents to regenerate the adsorbent. First, dye particles were adsorbed onto the Fe_3_O_4_@inulin. After the adsorption process, the dye-loaded particles of the adsorbent were regenerated via ethanol or acetone, exhaustively washed with DI water and then used for dye adsorption again. The Fe_3_O_4_@inulin was reused six times to check its applicability in removing MO and CV dyes. The reusability results for the adsorption of both dyes are reported in Fig. 7 under their optimum solution pH and adsorbent dosage. Notably, the removal efficiency of Fe_3_O_4_@inulin magnetic composite decreases from 94.92 to 83.34% and 91.13–78.86% after six successive adsorption cycles for MO and CV dyes, respectively, maintaining more than 87% of their initial adsorption rates. The decrease in the adsorption performance with increasing cycle number might be due to the saturation of sorption sites by strongly adsorbed dye molecules. The Fe_3_O_4_@inulin exhibits high MO and CV removal rates with excellent reusability.

Even though each sorbent has unique nature, intrinsic characteristics, advantages, and disadvantages, the adsorption capacity of the Fe_3_O_4_@inulin for MO and CV removal is compared with other bio-nano adsorbents. Table 4 compares different bio-adsorbents at their optimal solution pH. The best solution pH, isotherm and kinetics models are listed as well. As shown, the capacity of magnetized inulin is higher than that of similar magnetized biomaterials in removing both dyes. Such a performance approves that the Fe_3_O_4_@inulin is an excellent magnetized sorbent with good stability for adsorption of both anionic and cationic dyes under an appropriate solution pH.

**Table 4:** Performance comparison of different bio-nano adsorbents for treating water contaminated with MO and CV dyes.

Adsorbent	Dye	Solution pH	Isotherm model	Kinetics model	*Q*_*m*_ [mg/g]	Reusability (cycle and reduction in removal efficiency)	References
Chitosan microspheres	MO	3.1	Langmuir	PSO	207	5 and ~ 9%	^45^
Modified gum Tragacanth/graphene oxide	CV	8	Langmuir	PSO	94.0	3 and ~ 15%	^46^
Magnetic lignin-based carbon nanoparticles	MO	5	Langmuir	PSO	113.0	4 & 10%	^47^
Magnetic starch-graft-poly(acrylic acid) hydrogels	CV	7	Langmuir	PSO	80.64	5 and ~ 5%	^48^
Layered double hydroxides@Fe_3_O_4_/PVA	MO	6	Freundlich	PSO	19.59	4 and 50%	^49^
Magnetic kappa-carrageenan nanocomposite beads	CV	8	Langmuir	PSO	84.7		^50^
Xanthan gum/PVI hydrogel	CV	7	Langmuir	PFO	453.0		^51^
Ferric oxide-biochar nanocomposites	MO	8	Freundlich	PSO	20.53		^52^
Modified cellulose with glycidyl methacrylate	CV	9	Langmuir	PSO	218.8	8 and ~ 30	^53^
Chitosan/organic rectorite composite	MO	3	Langmuir	PSO	5.56		^54^
Hydroxyapatite nanoparticles impregnated magnetic bentonite	CV	8	Freundlich	PSO	1290.30	5 and ~ 5%	^55^
Fe_3_O_4_@inulin	MO	5	Langmuir	PSO	276.26	6 and ~ 11%	This work
	CV	7	Langmuir	PSO	223.57	6 and ~ 12%	

PVA: ed poly (vinyl alcohol), PVI: poly (N-vinyl imidazole).

## Conclusion

Nowadays, dye-contaminated water has become a severe environmental concern due to growing industrialization worldwide. Thus, environmentalists and researchers should urgently consider developing efficient methods and materials to mitigate such problems. In this work, we synthesized a new bio-nano adsorbent, i.e., magnetized inulin with Fe_3_O_4_, to remove toxic anionic and cationic dyes from wastewater. Various physical and structural analyses were conducted to evaluate the fabrication of the magnetic nanocomposite. According to the FTIR spectra, the presence of the Fe–O bond approved the successful synthesis of Fe_3_O_4_@inulin with an average crystalline size of 10.9 nm obtained from its XRD pattern. The SEM–EDX results demonstrated a relatively uniform, regular dispersion of spherical nanoparticles ranging from 10 to 20 nm on the composite surface. Based on the BET analysis, the adsorbent's specific surface area, pore volume, and average pore diameter were 66.15 m^2^/g, 0.181 cm^3^/g, and 11.027 nm, respectively. The VSM analysis showed that the nanocomposite's magnetic saturation, i.e., 40.21 emu/g, was about 34% lower than that for pure Fe_3_O_4_ nanoparticles. Considering the pH_PZC_ value of 6 and the anionic and cationic characteristics of both dyes, the optimal solution pH was 5 for MO and 7 for CV. According to the highest *R*^2^ value (> 0.99) and the lowest reduced chi-squared, the Langmuir isotherm fitted better the experimental data with a maximum adsorption capacity of 276.26 mg/g for MO and 223.57 mg/g for CV at 338 K. Considering these statistical criteria, the pseudo-second-order model (*R*^2^ > 0.99 and reduced chi-squared < 1) was a better kinetics model than pseudo-second-order and Elovich models in predicting the kinetics data of both dyes. In addition, the thermodynamic parameter demonstrated that the dye adsorption by Fe_3_O_4_@inulin was an endothermic and spontaneous process. Furthermore, after using the adsorbent six times, its removal efficiency maintained more than 87% of its initial adsorption rate for both pollutants, exhibiting excellent stability and reusability of the prepared nanocomposite. Overall, the comparison of the ability of different magnetized adsorbents in adsorbing cationic and anionic dyes verified that the Fe_3_O_4_@inulin could be a promising candidate for treating water contaminated with toxic dyes.

## Supplementary Information

Supplementary Figure S1.

## Data Availability

Data are available with the permission of [Prof. Ali Maleki]. The data that support the findings of this study are available from the corresponding authors, [Prof. Ali Maleki and Prof. Zhenjiang You], upon reasonable request.
