# Supplementary figures and images for: Magnetized inulin by Fe3O4 as a bio-nano adsorbent for treating water contaminated with methyl orange and crystal violet dyes
Source: Sci Rep. 2022 Dec 20;12:22034. doi: 10.1038/s41598-022-26652-7 (PMC9767922; doi:10.1038/s41598-022-26652-7)

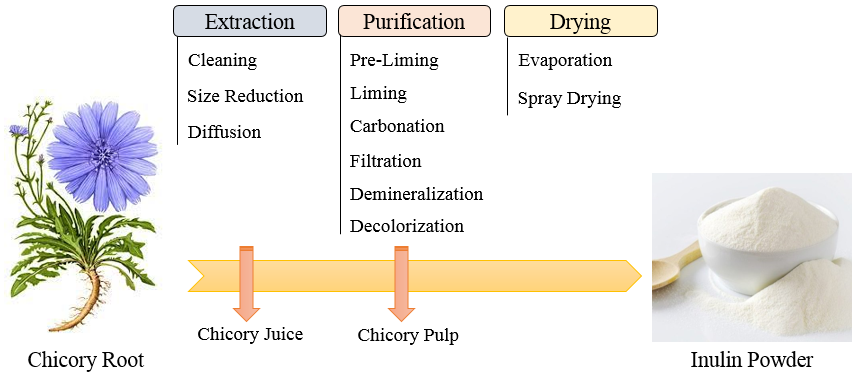


**Fig. S1.** Diagram of production process of inulin from chicory root.

Supplement: Supplementary file 1 — Supplementary Figure S1. [file 41598_2022_26652_MOESM1_ESM.docx]
